# Service use, healthcare costs and productivity losses of functional cognitive disorder in a sample of attenders to out-patient cognitive, neurology and memory clinics

**DOI:** 10.1192/bjo.2025.10766

**Published:** 2025-07-16

**Authors:** Barbara Barrett, Joan Agwuna, Sarah Cope, Mark J. Edwards, Norman Poole

**Affiliations:** Institute of Psychiatry, Psychology & Neuroscience, King’s College London, London, UK; South West London and St George’s Mental Health NHS Trust, London, UK; South London and Maudsley NHS Foundation Trust, London, UK

**Keywords:** Functional cognitive disorder, health economics, costs

## Abstract

**Background:**

People with functional disorders often receive high levels of healthcare resource use yet have poor outcomes. The health service costs and productivity losses of functional cognitive disorders (FCD) is unknown.

**Aims:**

This study aims to report the cost to health services and productivity losses of FCD.

**Method:**

Examination of healthcare service use and productivity losses in a sample of individuals with FCD who had attended a specialist out-patient clinic in south London.

**Results:**

The findings revealed high rates of healthcare use, including frequent contact with general practitioners, psychologists and hospital services, as well as work absences. The total estimated cost of healthcare and productivity losses per individual over 6 months was £1114.

**Conclusions:**

These results highlight the need for effective and efficient diagnostic pathways, targeted and effective interventions, and improved support for individuals with FCD.

Functional cognitive disorder (FCD) is a subtype of functional neurological disorder (FND). It is characterised by distressing and impairing cognitive symptoms, such as failure to retrieve over-learned memories (like names and bank PINs), with specific positive features that reveal intact memory (for instance, providing a detailed description of a memory lapse), and does not follow the typical pattern of recognised neuropathological conditions.^1^ Individuals with FCD experience considerable difficulties with memory and attention that significantly impact on their lives, but unlike other cognitive disorders, such as Alzheimer’s disease, cognitive function in FCD is stable and unfluctuating and is thought not to follow a progressive course,^2^ although long term follow-up studies are rare.

Health economists are motivated to study functional disorders because of the relationship between these conditions and high levels of healthcare service use. High levels of healthcare resource use and poor outcomes are suggestive of an inefficient allocation of resources since the opportunity cost suggests they could produce better outcomes elsewhere.^3^ A recent systematic review of FNDs described their substantial direct and indirect costs.^4^ The pathway to the diagnosis and treatment of functional disorders is often protracted, with the inclusion of potentially unnecessary tests and interventions, and a substantial delay between the onset of symptoms and diagnosis.^5^ It is possible that repeated diagnostic tests and out-patient appointments are a result of a lack of appropriate medical education and the stigma associated with the disorder.^6^ The flawed approach of seeking investigations to ‘rule out’ alternative diagnoses rather than making the diagnosis based on positive clinical signs and features, especially likely when diagnosing cognitive disorders, contributes to the substantial costs to health systems.^6^ Indeed, investigations to identify biomarkers of neurodegeneration may themselves trigger FCD^2^ through a nocebo response that is reinforced via attention, stress and anxiety. Alongside these healthcare costs, functional disorders can result in individuals missing work, withdrawing from employment and requiring formal or informal care, resulting in further economic impacts via productivity losses.^7^

While there is growing evidence of the costs to health services and productivity losses of other functional disorders, the health service and productivity costs of FCD are unknown. It is thought that the persistent unmet need and resistance to diagnosis leads to a cycle of referrals between primary and secondary care,^1^ but there is little evidence from primary data collection to corroborate this.

The purpose of this study is first to generate evidence on patterns of service use in FCD and second to estimate the healthcare and societal costs of individuals with FCD, in those attending an out-patient clinic. Evidence of the costs to health services and productivity losses of this disorder could contribute to the development of strategies for a more efficient allocation of scarce healthcare resources, as well as improvements in care pathways for those with FCD.

## Method

People attending neuropsychiatry, cognitive neurology and diagnostic memory out-patient clinics were recruited to the ACT4FCD study, a full description of which has been published.^8^ In brief, patients diagnosed with FCD by clinicians working in cognitive neurology, neuropsychiatry or diagnostic memory clinics were offered the opportunity to participate in a feasibility study of an online group delivering acceptance and commitment therapy intervention for their condition if they met the inclusion and exclusion criteria detailed in Box 1. Eighty-six potential participants were referred in to the trial of whom 44 (51%) were successfully recruited and randomised. Most participants were recruited from the neuropsychiatry clinic (*n* = 23; 52%); 14 (32%) came from cognitive neurology, while the fewest (*n* = 7; 16%) came from memory clinics, who were also the least likely to consent to randomisation.

Box 1 Inclusion/exclusion criteria

Inclusion criteria

A diagnosis of FCD established in clinic and confirmed via clinical notes and examination findings.

Aged 18 or over.

Able to provide written informed consent.

Exclusion criteria

Severe cognitive symptoms associated with a primary psychiatric disorder (e.g. depression, severe generalised anxiety disorder (GAD-7), post-traumatic stress disorder (PTSD), bipolar affective disorder, schizophrenia).

Considered at ‘medium’ or ‘high’ risk of deliberate self-harm and/or suicide (based on clinical assessment).

A coexisting functional disorder (e.g. functional seizures; comorbid functional diagnosis acceptable so long as those symptoms did not dominate the clinical picture).

Diagnosis of dementia.

Diagnosis of learning disability.

Insufficient command of English to engage in conversation without an interpreter.

The cost analysis was made from both a National Health Service (NHS) and a Personal Social Services (PSS) perspective, as required by UK decision-makers,^9^ and a societal perspective, to include productivity losses due to time off work. At baseline as well as a range of demographic and clinical measures, the Adult Service Use Schedule (AD-SUS) was also administered. The AD-SUS is a self-report questionnaire specifically designed for this study and adapted from previous work in similar populations.^10^ It is separated into four sections and refers to the 6 months preceding the day of completion. First, participants are asked about their employment status, and how much time they have taken off work due to their ill health. Second, the AD-SUS asks participants to recall the number of times they have seen a range of community-based health and mental health care services. Third, the questionnaire collects information on in-patient admissions, diagnostic tests, out-patient appointments and attendances at accident and emergency departments. Finally, patients are asked if they have been prescribed any psychotropic medication. Participants were able to complete the AD-SUS via an individualised weblink or a paper copy. Data were held on a REDCap database^11^ and backed up weekly on a secure server. The data collection and management was in line with GDPR Data Protection Act (2018) and South West London and St George’s Mental Health NHS Trust’s information governance.

The total cost of services for each participant was calculated by multiplying the quantity of each service by its unit cost for the financial year 2022–2023 and employment losses calculated using median earnings for the UK. Routine unit costs were employed, and these are detailed in Table 1. Mean average service use data and cost data are presented, alongside the proportion of the sample accessing each service at least once.

**Table 1:** Unit costs and sources

Cost description	Unit cost (£)	Source
Median earnings, per hour	12–17	(23)
In-patient stay, per night	985	(24)
Out-patient, per appointment	41–276	(24)
Diagnostics, per test	6–214	(24)
Accident and emergency, per visit	170	(24)
Ambulance, per call out	276	(24)
Medication, per month	3–43	(25)

## Results

Demographic characteristics are detailed in Table 2, and the employment status and types of health service contacts that study participants accessed are detailed in Table 3. In the 6-month period prior to completing the questionnaire, most participants reported that they spent at least 1 month working, either full-time (52%), or part-time (24%). At least a third had taken time off work due to ill health, for an average of 9 days. For primary healthcare, contact with general practitioners was common, with at least two-thirds of the participants having one or more contact. There was evidence of use of mental health services, with half of the study participants having seen a psychologist or a counsellor in the 6 months prior to interview. The most widely used services in secondary care were diagnostic tests (almost half with at least one contact) and out-patient appointments (nearly two-thirds with at least one contact). Around a quarter of the participants were on an antidepressant, with low use of other psychotropic medication.

**Table 2:** Sociodemographic information (*n* = 44)

	Mean (s.d.)
Age (years)	50.10 (12.87)
	*n* (%)
Gender	
Female	27 (61.4)
Male	17 (38.6)
Ethnicity	
Asian/Asian British	6 (13.6)
Arab/Arab British	1 (2.3)
Black/Black British	1 (2.3)
Dual Heritage	2 (4.5)
White	34 (77.3)
White British	25 (56.8)
White Irish	2 (4.5)
White Other	7 (15.9)
Employed	26 (59.1)
Full-time employment	17 (38.6)
Part-time employment	9 (20.5)

**Table 3:** Use of services over 6 months prior to interview (*n* = 42)

Service (unit)	Mean (s.d.)	%
Employment		
Full time (months)	2.74 (2.83)	52
Part-time (months)	1.31 (2.44)	24
Unemployed (months)	1.90 (2.71)	38
Education/training (months)	0.36 (1.19)	12
Absent due to ill health (days)	9.64 (25.27)	40
Primary healthcare		
General practitioner (number)	2.69 (3.43)	71
Practice nurse (number)	0.38 (0.79)	29
Community mental health team (number)	0.81 (2.02)	24
Psychologist/counsellor (number)	2.52 (5.11)	50
Physiotherapist/occupational therapist/speech and language therapist (number)	2.29 (5.53)	26
National Health Service 111 (number)	0.29 (0.74)	19
Secondary healthcare		
In-patient stay (nights)	0.10 (0.30)	10
Accident and emergency (attendances)	0.21 (0.56)	14
Ambulance (call outs)	0.02 (0.15)	2
Diagnostic tests (number)	2.07 (3.37)	48
Out-patient appointments (number)	2.86 (2.41)	62
Medication		
Antidepressant		26
Sleeping tablet		10
Anti-anxiety		17
Mood stabiliser		5
Attention-deficit hyperactivity disorder medication		0

Total costs over 6 months to health and social care services were on average £1045, with an equal split between primary and secondary care services (Table 4). Productivity losses were on average £69 (the cost of time off work for those in employment), generating a societal total cost estimate of £1114.

**Table 4:** Total costs over 6 months prior to interview (*n* = 42)

Baseline	Mean (s.d.)	
Primary healthcare	529.98 (833.99)	
Secondary healthcare	507.14 (625.29)	
Medication	7.69 (0.58)	
**Total cost, health and social care**	**1044.82 (1416.89)**	
Productivity losses	69.42 (54.37)	
**Total societal cost**	**1114.23 (1142.80)**	

## Discussion

The findings of this study provide useful insight into the health service use of people with FCD and their time off work due to ill health. The findings reflect research from our group on the lived experiences of those with an FCD diagnosis who reported contacting a range of clinicians prior to diagnosis and being ‘bounced’ between different healthcare professionals.^12^

Over 90% of the participants were of working age, and 52% spent at least 1 month in full-time employment, with a further 24% with at least 1 month in part-time work. While this suggests that there is a proportion of people with FCD who are economically inactive, employment rates are higher than those found for other functional neurological disorders. In comparable UK samples, O’Connell and colleagues found employment rates of around 25%,^13^ and O’Mahony reported full-time employment at 17% and part-time employment at 5.2% in participants recruited from similarly specialist services.^14^ This suggests that those diagnosed with FCD may have less disability when compared with patients diagnosed with other FND presentations.

General practitioners were widely accessed by patients, suggesting they were seeking help for their symptoms, further investigations or treatment of comorbidities. In the general population, rates of use of NHS talking therapies is around 2%,^15^ so the 50% reported rates of contact with psychology and counselling in this sample is much higher, suggesting that participants in the study experienced psychological distress for which they were seeking treatment. This suggests a high psychological impact of the symptoms. Similarly, use of antidepressants at 26% accords with the known association between anxiety and mood disorders with FCD,^16^ and which is substantially higher than prescribing rates of 16% in the general population.^17^

The secondary care service use reflects the help seeking often associated with functional disorders, with nearly half of participants having a hospital-based diagnostic and two-thirds an out-patient appointment (recruitment to the study was both from out-patient clinics and a list of those willing to be contacted for research studies kept by the cognitive neurology service). Despite this, as we have reported elsewhere,^12^ as with other functional disorders, there are high levels of felt stigma and dissatisfaction with healthcare as even once a diagnosis has been made little or no active interventions are offered.^18^ It is likely that numbers with the disorder could increase with the availability of pre-symptomatic diagnoses using biomarkers,^19^ and potential expansions in the diagnostic criteria, as well as novel new treatments, such as the monoclonal antibody Lecanemab.^20^

The total cost to NHS/PSS was on average £1044 over 6 months, which is lower than the healthcare costs of people with FND in general, which was substantially higher, at £3229 over 6 months, in research which used similar methods to the collection of service use data.^14^ This implies that FCD symptoms can potentially have less of an impact on functioning when compared with other FND presentations, but this warrants further investigation with a larger sample size. It is also difficult to assess given that patients may have other FND conditions and comorbidities.

It is important to acknowledge the limitations of this study. The sample size was relatively small and recruited from secondary care clinics which assess patients with memory symptoms, so the findings may not be generalisable to the entire population with FCD. We were not able to include estimates of the costs of informal care, which previous papers have suggested might be substantial in similar groups,^14^ nor did we capture the impact of welfare claims, which studies in similar populations have shown can be considerable.^7^ However, it remains the largest sample of FCD in the UK, and we believe that the methods to collect service use data and estimate costs were rigorous.

In conclusion, this study adds to recent research demonstrating elevated healthcare costs are associated with functional disorders. It supports the shift from ‘rule out’ to ‘rule in’ diagnosis in FND generally,^21^ which has the potential to save costs via a reduction in referrals and complex diagnostics, and increase confidence in diagnostic accuracy while reducing stigma and dissatisfaction. This has been argued for elsewhere^2^ but has yet to be properly implemented for FCD. Patients, however, also deserve active treatment, such as psychological interventions, that has the potential to reduce symptom burden and distress^22^ while improving the quality and productivity of their lives.

## Data Availability

The data that support the findings of this study are available on request from the corresponding author, B.B.
